# Gastric foveolar adenoma in the duodenal bulb: case report

**DOI:** 10.11604/pamj.2024.48.178.43852

**Published:** 2024-08-14

**Authors:** Walid El Ouardi, Bochra Bouchabou, Asmaa Regragui, Mustapha Benazzouz

**Affiliations:** 1 Department of Gastroenterology, Faculty of Medicine and Pharmacy, Mohammed V University, Rabat, Morocco,; 2 Department of Hepatogastroenterology, Mongi Slim Hospital, Tunis, Tunisia,; 3 Department of Anatomopathology, Pathological Anatomy Center of Agdal, Rabat, Morocco,; 4 Department of Gastroenterology, International University of Rabat, Riad Annakhil International Polyclinic, Rabat, Morocco

**Keywords:** Duodenal bulb, polyp, gastric foveolar adenoma, case report

## Abstract

Foveolar-type adenomas are very rare lesions, representing approximately 2.7% of duodenal adenomas with gastric phenotype, histologically characterized by tall columnar cells resembling gastric foveolar epithelium and a tubulovillous structure with various degrees of dysplasia. Their risk of progression to adenocarcinoma is related to the size of the polyp and the presence of high-grade dysplasia. The recommended therapeutic approach is the endoscopic resection. our clinical case reports a rare case of a patient in whom gastric foveolar adenoma was incidentally discovered as an 8 mm sessile polyp in the duodenal bulb resected entirely by en-bloc mucosectomy technique. Through this case, we draw attention to the existence of bulbar adenomas, which carry a risk of dysplasia and progression to adenocarcinoma.

## Introduction

Duodenal adenomas with gastric phenotype are subclassified into pyloric gland adenoma and foveolar adenoma [1]. Foveolar-type adenomas are very rare lesions, representing approximately 2.7% of duodenal adenomas with gastric phenotype and are preferentially located in the proximal duodenum having a risk of dysplasia and progression to adenocarcinoma [1]. We report a case of an 8 mm polyp in the duodenal bulb histologically corresponding to gastric foveolar adenoma with low-grade dysplasia.

## Patient and observation

**Patient information:** an 86-year-old white women patient from north Africa, non-smoker, non-alcoholic, with a medical history of hypertension and depression controlled under medication, and a uterine fibroid surgery performed 24 years ago. She consulted for asthenia.

**Clinical findings:** at the presentation there was no signs of gynecological or digestive bleeding nor any other sign of bleeding. The physical examination reveals a patient in good general condition, body mass index at 22 kg/m, hemodynamically stable with no cutaneous and mucous pallor. Digital rectal examination and vaginal examination were normal.

**Timeline of current episode:** October 2023: onset of symptoms, November 2023: completion of the biological assessment, December 2023: upper and lower gastrointestinal endoscopy.

**Figure 1:**
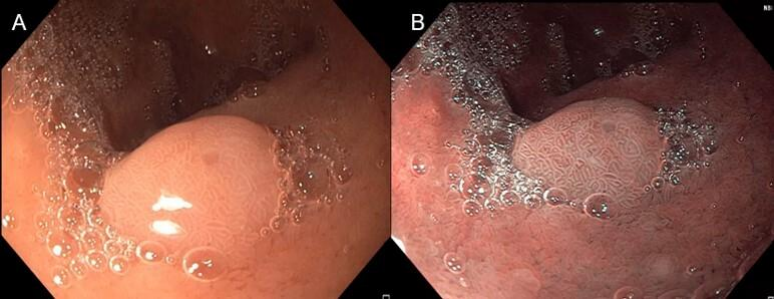
polyp in the duodenal bulb: A) in white light; B) narrow band imaging

**Figure 2:**
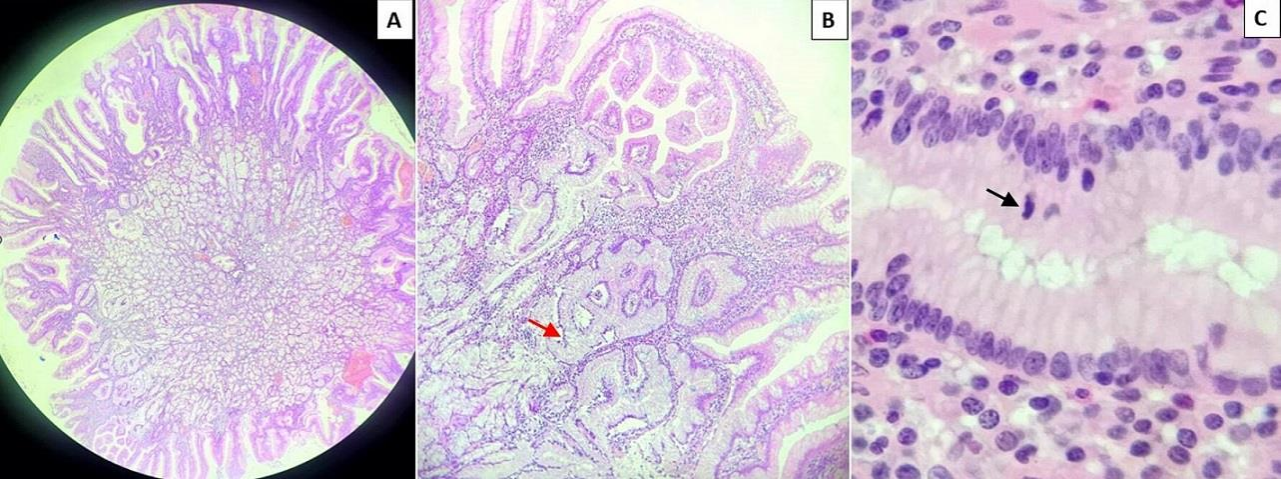
histopathology of the polyp: A) image of the polyp in its entirety with hyperplastic foveolar border (hematoxylin and eosin x40); B) hypersecretory foveolar proliferation invaginating into the chorion (red arrow) (hematoxylin and eosin x100); C) hypersecretory foveolar border with discrete pseudostratification, mild atypia, and a few mitoses (black arrow) (hematoxylin and eosin x400)

**Diagnostic assessment:** biology assessment reveal hypoferritinemia at 9 ng/ml with normal hemoglobin level. The upper gastrointestinal endoscopy reveals, incidentally, an 8 mm sessile polyp at the duodenal bulb. Its color is indifferent compared to the adjacent mucosa, and the pit pattern reveals elongated regular crypts with branching, creating a cerebriform appearance with regular vessels surrounding the glands, classified as Paris Is, Kudo IV, Sano II (Figure 1) suggesting the appearance of an adenomatous polyp in the duodenal bulb. Histopathology reveals gastric mucosa with adenomatous proliferation composed of cubic epithelial cells with rounded nuclei and a discreet increase in the nuclear-cytoplasmic ratio without significant mitotic activity. These structures border the surface lining and invaginate deeply, forming tubular structures, indicative of a gastric foveolar type adenoma with low-grade dysplasia (LGD) (Figure 2). The rest of the gastroscopy showed no notable anomalies. The ileocolonoscopy and small bowl video capsule endoscopy were normal. For the hypoferritinemia, no digestive cause was found, and the patient was referred for gynecological evaluation.

**Diagnosis:** the final diagnosis was the incidental finding of an 8 mm polyp corresponding to a gastric foveolar type adenoma with low-grade dysplasia located in the duodenal bulb.

**Therapeutic interventions:** the polyp was completely resected, and no further treatment was considered.

**Follow-up and outcome of interventions:** the post-procedure course was uneventful.

**Informed consent:** informed written consent was obtained from the patient.

## Discussion

Duodenal polyps are typically categorized based on their location, whether situated in the duodenal bulb, ampullary/peri-ampullary region, or the distal duodenum. Some specific lesions are predominantly identified in the ampullary/peri-ampullary region, such as tumors demonstrating pancreatobiliary differentiation, gangliocytic paragangliomas, and neuroendocrine tumors expressing somatostatin. The majority of duodenal polyps are non-neoplastic, representing regenerative/hyperplastic nodules of foveolar epithelium or proliferations of Brunner glands (38%). Less common types of polyps include heterotopic lesions (6%) and neoplastic lesions (11%) [2]. At present, duodenal adenomatous polyps are classified according to the mucin phenotype into intestinal (89.1%) and gastric type (10.9%). The intestinal-type polyps are morphologically subdivided into tubular and tubulovillous adenomas and the gastric-type into pyloric gland adenomas (8%) and foveolar adenomas (3%) [1,3]. Relatively high proportion (approximately 60%) of non-ampullary duodenal adenomas are associated with FAP (Familial adenomatous polyposis) or MAP (MUTYH-associated polyposis). Non-syndromic non-ampullary adenomas are found in only 0.3-0.5% [4].

Foveolar-type adenomas are very rare lesions, representing approximately 2.7% of duodenal adenomas with gastric phenotype. Endoscopically these lesions are polypoid and have a median tumor diameter of 9.7 mm (range: 8-20 mm) [1], Mitsuishi *et al*. [3] and Vieth *et al*. [5] have shown that these lesions mainly develop in the first portion of the duodenum. Histologically, foveolar-type adenoma was characterized by tall columnar cells resembling gastric foveolar epithelium and a tubulovillous structure with various degrees of dysplasia [2]. All gastric-type adenomas classified with hematoxylin and eosin staining showed an intense MUC5AC-positive gastric foveolar-like cell component. In addition, MUC6 immunostaining was useful for subclassifying gastric-type adenomas into a pyloric gland type and a foveolar type [6]. Okada *et al*. conducted an endoscopic follow-up study on histologically confirmed low-grade duodenal adenomas. Their findings revealed that out of 43 cases, 9 (20.9%) exhibited advancement to high-grade dysplasia, including two cases of non-invasive carcinomas. These studies indicate the potential for certain duodenal adenomas to evolve into adenocarcinomas with an adenoma-carcinoma sequence similar to that recognized in colon cancer. Okada *et al*. reported in their study that histological high-grade dysplasia (HGD), identified through multivariate analysis, is a significant predictor of progression to adenocarcinoma [7], in addition to the HGD the risk of neoplastic progression appears to be related to the size, then lesions with LGD and < 20 mm in size presents a low risk of progression to adenocarcinoma (4.7%) [2].

The recommended approach for removing small, sporadic non-ampullary duodenal adenomas (<10 mm) is cold-snare polypectomy. Larger lesions (>10 mm), which are more likely associated with high-grade dysplasia or adenocarcinoma, can be effectively excised using endoscopic mucosal resection (EMR). However, it is not advisable to employ endoscopic submucosal dissection due to the relatively thin and vascular nature of the duodenal wall, which poses a relatively high risk of perforation (up to 30%) [8].

For the prognosis, Mitsuishi *et al*. showed that regardless of the grade of atypia and mucin phenotypes, lesions were completely resected in all cases, and no cases had recurrence or progression [3]. It is known that patients with sporadic duodenal adenoma are typically comorbid with colorectal neoplasia [9]. Hijikata *et al*. [6] showed that all duodenal adenomas that were comorbid with colorectal neoplasms were the intestinal type.

## Conclusion

We report in our case report a case of a gastric foveolar type adenoma located in the duodenal bulb and draw attention to the fact that the duodenal bulb can be the site of adenomatous polyps with varying degrees of dysplasia, potentially progressing to adenocarcinoma.
